# CVD Growth of Hematite Thin Films for Photoelectrochemical Water Splitting: Effect of Precursor-Substrate Distance on Their Final Properties

**DOI:** 10.3390/molecules28041954

**Published:** 2023-02-18

**Authors:** Leunam Fernandez-Izquierdo, Enzo Luigi Spera, Boris Durán, Ricardo Enrique Marotti, Enrique Ariel Dalchiele, Rodrigo del Rio, Samuel A. Hevia

**Affiliations:** 1 Centro de Investigación en Nanotecnología y Materiales Avanzados (CIEN-UC), Pontificia Universidad Católica de Chile, Casilla 306, Santiago 6904411, Chile; 2 Facultad de Química y de Farmacia, Pontificia Universidad Católica de Chile, Casilla 306, Santiago 6904411, Chile; 3 Department of Material Science and Engineering, The University of Texas at Dallas, 2601 North Floyd Road RL10, Richardson, TX 75080, USA; 4 Instituto de Física, Facultad de Ingeniería, Universidad de la República, Julio Herrera y Reissig 565, C.C. 30, Montevideo 11000, Uruguay; 5 Departamento de Medicina Traslacional, Facultad de Medicina, Universidad Católica del Maule, Talca 3480112, Chile; 6 Instituto de Física, Pontificia Universidad Católica de Chile, Casilla 306, Santiago 6904411, Chile

**Keywords:** hematite, chemical vapor deposition, thin film, water splitting

## Abstract

The development of photoelectrode materials for efficient water splitting using solar energy is a crucial research topic for green hydrogen production. These materials need to be abundant, fabricated on a large scale, and at low cost. In this context, hematite is a promising material that has been widely studied. However, it is a huge challenge to achieve high-efficiency performance as a photoelectrode in water splitting. This paper reports a study of chemical vapor deposition (CVD) growth of hematite nanocrystalline thin films on fluorine-doped tin oxide as a photoanode for photoelectrochemical water splitting, with a particular focus on the effect of the precursor–substrate distance in the CVD system. A full morphological, structural, and optical characterization of hematite nanocrystalline thin films was performed, revealing that no change occurred in the structure of the films as a function of the previously mentioned distance. However, it was found that the thickness of the hematite film, which is a critical parameter in the photoelectrochemical performance, linearly depends on the precursor–substrate distance; however, the electrochemical response exhibits a nonmonotonic behavior. A maximum photocurrent value close to 2.5 mA/cm^2^ was obtained for a film with a thickness of around 220 nm under solar irradiation.

## 1. Introduction

Hematite is an attractive semiconductor material for photoelectrochemical and photocatalytic purposes due to its stability, abundance, and environmental compatibility, as well as its suitable bandgap and valence band edge position [1,2,3]. Moreover, this oxide shows a high potential for commercial application in water splitting since its theoretical solar-to-hydrogen efficiency (STH) is as high as 12.9% [1,3,4]. In general, hematite exhibits an n-type semiconducting behavior, which can be due to the tendency of hematite to become oxygen-deficient, irrespective of the preparation method [1]. Unfortunately, because hematite presents a weak optical absorption coefficient, small carrier mobility, and a short hole diffusion length, the illuminated hematite electrodes normally exhibit poor efficiency as photoanodes in water oxidation [5,6]. However, many studies have indicated that nanocrystalline morphology could be one way to overcome those limitations and increase the photon-to-current yield by minimizing the distance that minority carriers must diffuse before reaching the interface and contacts [1,7,8,9,10]. In addition, the large surface area exhibited by the nanostructured semiconductors is important in photoelectrochemical devices due to the large number of catalytic sites [3,7,11,12,13]. Additionally, the nano structuration facilitates the incorporation of other nanomaterials or atoms that improve the catalytic performance [14,15,16,17,18]. Several techniques have been investigated for the synthesis of hematite nanocrystalline thin films: thermal evaporation [19], aqueous chemical growth [20], spray-pyrolysis [10,21,22], sol-gel [23], and electrodeposition methods [1,8,13]. A low-cost and solvent-free method for preparing semiconducting thin films well-suited for the preparation of nanostructures is chemical vapor deposition (CVD) [24,25]. In the last years, several research groups have employed the CVD technique to obtain iron oxide thin films. Thermal CVD synthesis of α-Fe_2_O_3_ thin films by using different iron precursors such as carbonyls, alkoxides, and β-diketonates has been reported [24,25,26,27,28,29,30,31]. However, it is still necessary to achieve a deep understanding of the hematite thin film growth by CVD in order to develop efficient photoelectrodes for solar photoelectrochemical water splitting.

This work reports a study of the CVD growth process of hematite thin films onto fluorine-doped tin oxide (FTO) substrates by using the ferrocene organometallic compound as an iron precursor. A complete morphological and structural characterization of the hematite films was carried out using scanning electron microscopy (SEM), energy dispersive X-ray spectroscopy (EDS), Raman spectroscopy, X-ray photoelectron spectroscopy (XPS), and X-ray diffraction (XRD). An optical characterization with a thorough analysis was performed due to the importance of this property for the previously mentioned application. Finally, the photoelectrochemical response of the films was evaluated, and the dependence of the distance between the precursor and the substrates in the CVD system was studied in order to find the optimal fabrication condition that maximized the photoelectrochemical response.

## 2. Results and Discussion

### 2.1. Morphological and Structural Characterization of the Hematite Thin Films

**Figure 1:**
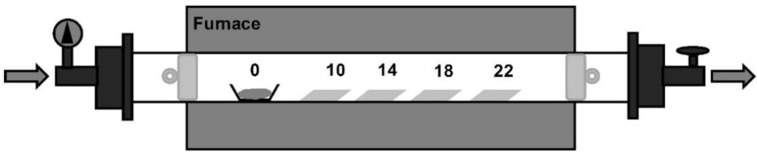
Scheme of the CVD system. Two quartz plugs were placed inside the quartz tube to establish the reaction zone. At the gas entrance (from left to right), was placed a quartz plug, at 10 cm from it was placed the boat with the precursor. The FTO substrates were located 10, 14, 18, and 22 cm from the boat. In the gas exit, a second plug was located (12 cm away from the last substrate). It is important to notice that the plugs do not seal the tube, they only obstruct the flow out from the reaction zone.

Nanostructured hematite thin films on FTO substrates were obtained by CVD using ferrocene as precursor in a two-step method. The substrates were located at 10, 14, 18, and 22 cm from the source of the precursor, as shown in Figure 1.

**Figure 2:**
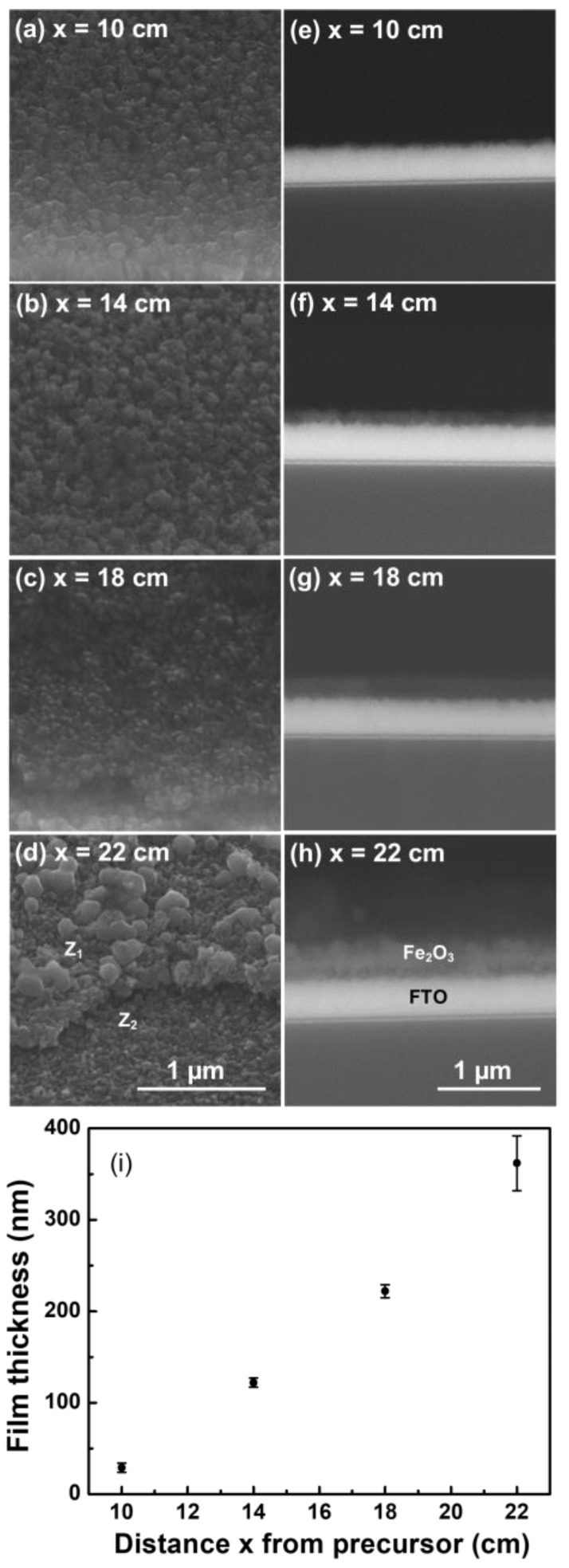
(**a**–**d**) correspond to 45-degree tilted view SEM micrographs of samples prepared at the positions 10, 14, 18, and 22 cm, respectively. In (**d**), Z_1_ and Z_2_ denote two different zones of the sample, the zone with hematite and the zone of bare FTO, respectively. (**e**–**h**) correspond to cross-section SEM micrographs of the same set of samples. (**i**) Plot of the film thickness (determined by the cross-section micrograph) as a function of their distance to the precursor.

It was observed that samples decreased in transparency at the same time as the distance of the precursor, called “x”, increased (see Figure 1). Differences in color and transparency of the electrodes can be observed in Figure S1 in the Supplementary Materials. Figure 2 shows SEM images of hematite films grown by CVD on FTO substrates at different values of position X. Figure 2a–d corresponds to 45-degree tilted view micrographs of samples prepared at the positions of 10, 14, 18, and 22 cm, respectively. Figure 2e–h corresponds to cross-section micrographs of the same set of samples. From this set of images, it is observed that the thickness and morphology of the film depend on the position of the substrate in the CVD system.

The morphology of the film consists of nanostructures joined to each other, giving the impression that growth in the form of bunches, where the upper ends could have the same origin. So, it could be possible to note that the growth mechanism followed a similar behavior to the one proposed in the Stranski–Krastanov model, where the interaction of the adsorbed atoms among them was similar to that of the adsorbed atoms with the substrate surface [9,32]. In this case, after forming one or more monolayers, subsequent layer growth became unfavorable, and islands were formed [33].

From the cross-section SEM micrographs (Figure 2e–h), it is observed that the thickness of the hematite films rises as the distance between the substrate and the precursor increase. By measuring the different thicknesses in the cross-section micrographs, it was observed that the FTO has a thickness of 315 nm, and the hematite films have thicknesses of 362, 222, 122, and 29 nm for the electrodes prepared at the positions of 10, 14, 18, and 22 cm, respectively. The plot of the film thickness as a function of their distance to the precursor is shown in Figure 2i. The hematite film thickness depends almost linearly on the precursor–substrate distance. The reason for this is that the employed CVD reactor included two quartz plugs, one at the entrance and the other at the exit of the tube reactor (see Figure 1). The purpose of these quartz plugs was to maintain the precursor confined inside the reaction chamber in the zone where the temperature is uniform. However, the presence of these plugs, particularly the outlet plug, jams the gas flow outlet, resulting in an increasing precursor concentration towards the end of the reactor tube. As a consequence of that, the thickness of the grown films follows this concentration gradient.

The dispersive energy X-ray spectroscopy (EDS) analysis, performed under the same conditions for all samples, showed mainly that the percentage of iron in the film rose as their thickness increased (Table S1 in the Supplementary Materials).

**Figure 3:**
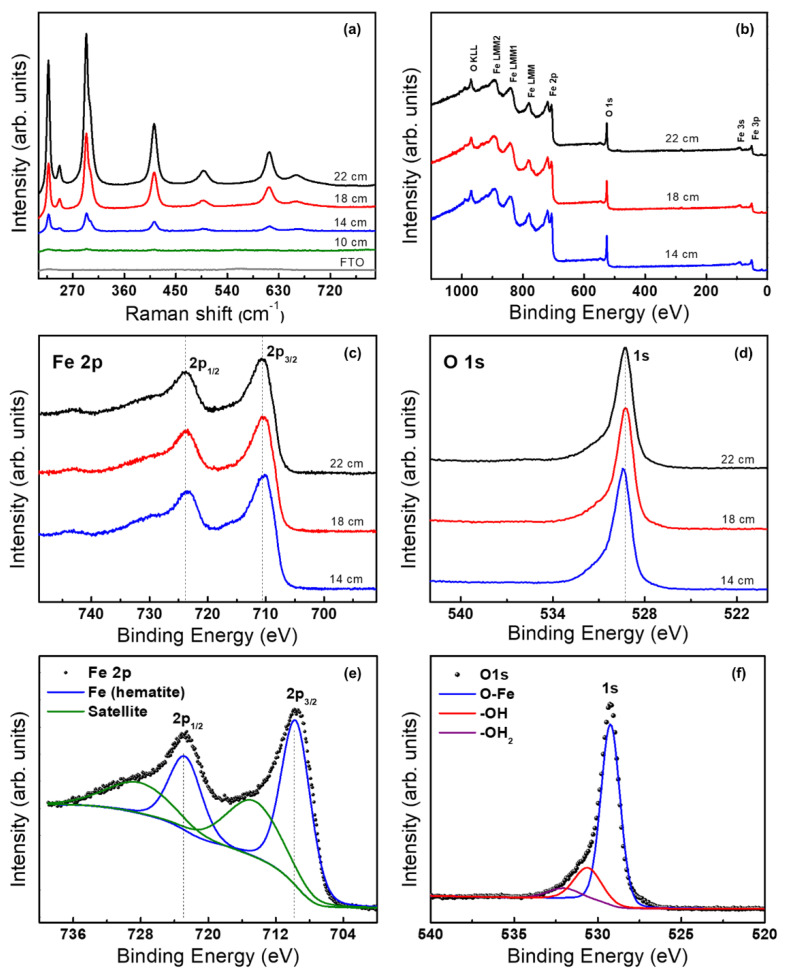
(**a**) Raman spectra of FTO and electrodes prepared at 10, 14, 18, and 22 cm. XPS spectra of hematite films grown at 14, 18 and 22 cm: (**b**) survey, (**c**,**d**) are the high-resolution measurements of signals Fe2p and O1s, respectively. (**e**,**f**) are the Fe2p and O1s signals of the film grown at 18 cm, fitted using the software Multipack.

Raman spectroscopy was performed in the range of 150–900 cm^−1^, where the iron oxides and the hematite exhibit their characteristic vibration modes. Figure 3a shows the Raman spectra of the hematite films prepared at 10, 14, 18, and 22 cm, and of the FTO substrate. Seven main resonances can be observed in the hematite film spectra with intensities that increase at the same time that the thickness of the film increases. The peaks located at 227 cm^−1^ and 498 cm^−1^ were assigned to the A_1g_ vibrational modes, and the peaks in 247, 294, 301, 412, and 613 cm^−1^ were assigned to the *E*_g_ vibrational modes of the hematite phase of iron oxide [19,26,34]. This analysis confirmed that the annealing treatment for 30 min at 550 °C was suitable to fully transform the initial iron precursor films to hematite. The peak at 660 cm^–1^ has been previously found in hematite. Initially, some researchers attributed this peak to the maghemite or magnetite presence. However, recent publications showed that this resonance has its origin in a Raman-forbidden LO Eu mode, activated through disorder-induced symmetry breaking. This structural disorder can be found in nanostructured systems, or in our case, in nanocrystalline thin films [35].

XPS spectra of the hematite films grown at 14, 18, and 22 cm are shown in Figure 3b. The signals associated with Fe and O are clearly identified in the spectra, and with lower intensity, the adventitious carbon is also present. High-resolution measurements of Fe2p and O1s are shown in Figure 3c,d, respectively. The shape of these signals is very similar between the films. Figure 3e,f presents the Fe2p and O1s signals of the film grown at 18 cm and fitted using the software Multipack (the fits for the other films are shown in Figure S2). The binding energy (BE) of peaks associated with Fe 2p^1/2^ and Fe 2p^3/2^ are 724.5 and 710.9 eV, respectively. These BE values are characteristic of the Fe(III) in hematite, which is also consistent with the BE of the peak associated with the Fe(III)–O bond in the signal O1s, which is 529.3 eV [3,23,36]. The Raman and XPS results confirm that the films are composed of hematite.

**Figure 4:**
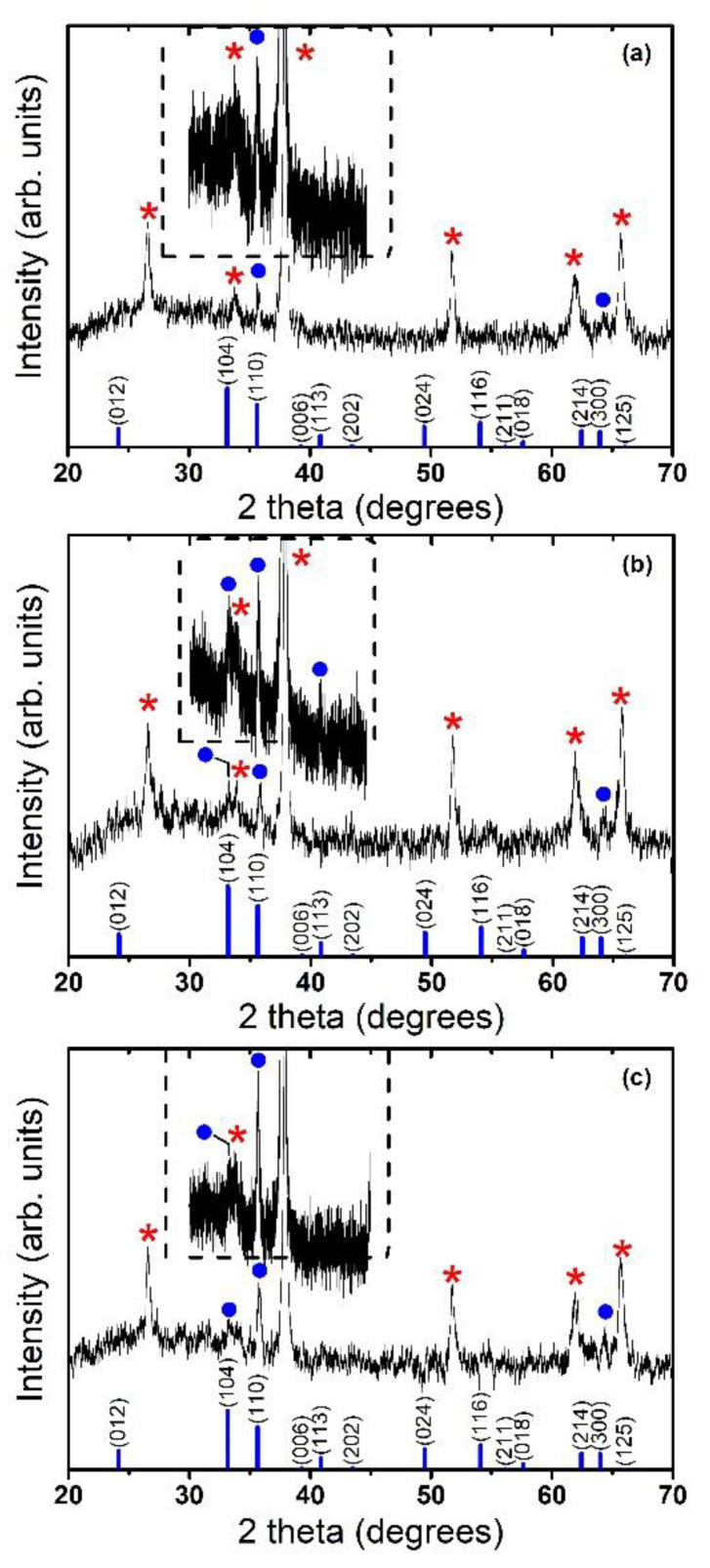
X-ray diffraction patterns of hematite films grown at (**a**) 14 cm, (**b**) 18 cm, and (**c**) 22 cm. JCPDS pattern of rhombohedrally centered hexagonal structure of Fe_2_O_3_ (α-Fe_2_O_3_, hematite), labeled with the corresponding crystallographic planes is also shown for comparison at the bottom of each panel. A zooming of the XRD pattern in the 2 theta range from 30 to 45 degrees is depicted as an inset in each panel. (●) and (*) symbols indicate the diffraction peaks originated from the hematite phase and from the SnO_2_:F substrate, respectively.

In order to study the structural properties of the hematite films, X-ray diffraction experiments have been carried out. Figure 4 shows the X-ray diffraction patterns of films prepared at positions 14, 18, and 22 cm. It can be seen that all diffraction peaks (except the peaks of the substrate), can be indexed to the rhombohedrally centered hexagonal structure of Fe_2_O_3_ (α-Fe_2_O_3_, hematite), which are in agreement with standard reported values [37], see JCPDS pattern at the bottom of each panel in Figure 4. All samples are polycrystalline, and the broadening of the diffraction peaks demonstrates the nanocrystalline character of nanostructured α-Fe_2_O_3_ layers. An average crystallite size could be obtained using the Scherrer formula for the crystallite size broadening of diffraction peaks:

(1) $$D=\frac{k\lambda }{\beta cos\theta }$$

where *k* = 0.94, *λ* is the X-ray wavelength, *θ* is the Bragg angle, and β is the FWHM of the diffraction peak [38]. By applying the above-mentioned Scherrer equation, typical crystallite size values of ca. 25 nm were estimated from (110) diffraction peak for the hematite films, irrespective of the position in the CVD reactor. Moreover, it can be appreciated in the XRD diffraction patterns depicted in Figure 4, that in contrast to the powder diffractogram α-Fe_2_O_3_ JCPDS pattern, the relative intensity of the (110) plane, is anomalous with respect to the other planes, evidencing a preferred crystallographic orientation along the (110) direction axis vertical to the substrate [5]. Therefore, this indicates that the hematite films grow along the (110) crystallographic direction (energetically most favorable [39]), that is, the growth axis is along the *c* direction [40]. It is worth mentioning that this preferential orientation is the best one for the hematite structure with good electrical conductivity [5], and it is good for the photoelectrochemical process, where the electron can flow through the (001) basal plane (due to anisotropic conductivity of hematite iron oxide [6]), to the back contact, and the hole can still hop laterally between (001) planes to reach the electrolyte interface [2,5].

### 2.2. Optical Characterization of the Hematite Thin Films

**Figure 5:**
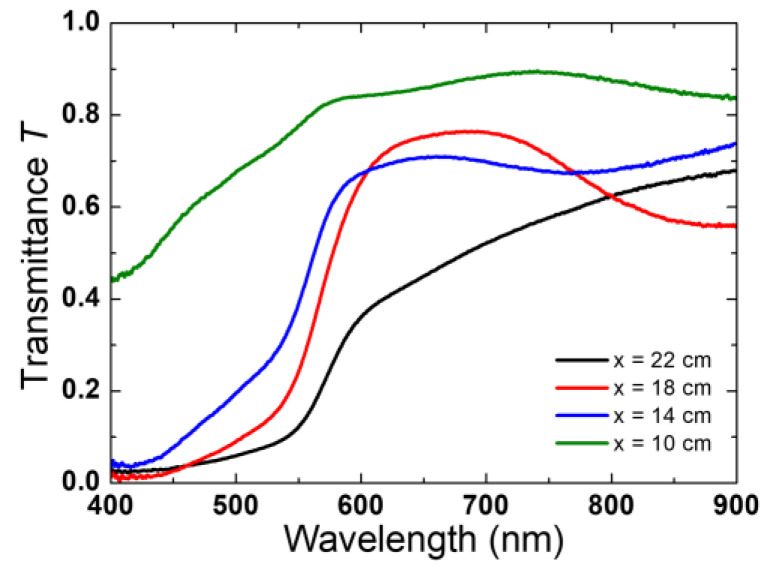
Optical transmittance of hematite films grown by CVD on FTO substrates. The spectra correspond to samples prepared at the positions of 10, 14, 18, and 22 cm.

As is shown in Figure S1 in the Supplementary Materials, all samples have a brick-red color, with the sample becoming less translucent as the distance to the precursor increases. The optical transmittance T spectra are shown in Figure 5. For all samples, except x = 10 cm, the transmittance is lower than 25% for wavelengths shorter than 500 nm and has an abrupt increase between 500 and 600 nm. This spectral feature is characteristic of an absorption edge; the position of this edge depends on the sample. A redshift of this absorption edge can be seen at the same time the x value increase. The sample x = 18.0 cm has a decrease in the transmittance after the absorption edge. It may originate in interferences by reflections at the film–air and film–substrate interfaces. The other samples have smaller variations in the transmittance, like oscillations, that could also be because of interferences by reflections.

To study the absorption edge, it is necessary to find the optical band gap *E*_g_. For this optical gap determination, the nature of the absorption edge must be assumed. This is because *E*_g_ values are obtained from plots of (α *h*ν)*^m^* vs photon energy *h*ν where the absorption coefficient α is estimated from the transmittance as α ~−ln*T* [41]. For direct transitions, the plot with *m* = 2 shows a linear region [42]. For indirect transitions, the plot will show a linear region for *m* = 1/2 [43]. In both cases, the corresponding *E*_g_ is obtained by extrapolating the linear fitting from the plot and finding the energy value where this linear fitting intersects the zero line. The lowest bandgap of α-Fe_2_O_3_ is usually reported to be around 1.9–2.2 eV [7,8,10,13,22,34,36,44,45,46,47,48,49,50,51,52,53,54]. However, while many authors address the corresponding transition to be indirect [13,44,46,47,54], others found direct transitions corresponding to the same edge [7,22,34,49,51,53]. As a way of comparing the results to previous works, several authors report both direct and indirect *E*_g_ values from different linear fittings made in the same spectral regions [8,50].

**Figure 6:**
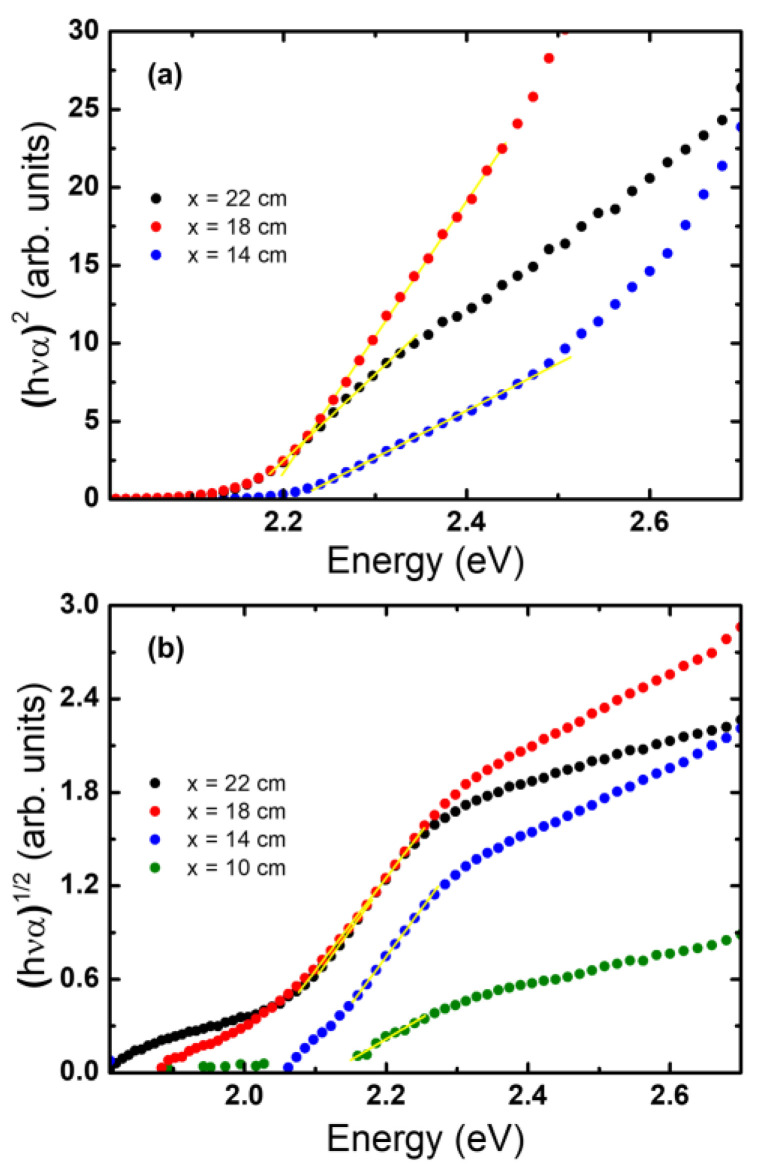
Direct (**a**) and indirect (**b**) optical bandgap of the hematite films. The Tauc plot corresponds to samples prepared at the positions of 10, 14, 18, and 22 cm.

Nevertheless, the absorption coefficient is the sum of all processes occurring in the sample. It has contributions of absorption because of amorphous phases, light dispersion, and reflections on the interfaces. All these processes introduce uncertainty in the zero-absorption line. Because of this, a correction of the absorption coefficient is made. The correction method used is like the one used in nanostructured composite materials [11,12,55,56]. It consists of subtracting an amorphous-like background absorption coefficient (α_back_) from the measured absorption coefficient (α_exp_) [43]. This background is obtained from a linear fitting in the transparency region of the (α_exp_ *h*ν)^1/2^ vs. *h*ν plot, where it should go to zero. After this correction, either a direct or an indirect optical gap can be estimated more precisely from α_corr_ = α_exp_ − α_back_ using the same method described before. The results obtained with this method can be seen in Figure 6a,b. For either case, the same α_corr_ was used. In Figure 6a the value of *E*_g_ is estimated, assuming that the gap is direct, and in Figure 6b, is estimated assuming the gap is indirect. The obtained values are summarized in Table 1. All values are within the usually reported range of 1.9–2.2 eV [7,8,10,13,22,34,36,44,45,46,47,48,49,50,51,52,53,54].

Comparing the values of both determinations, in all cases, the energy gap is lower when it is calculated as an indirect edge. The difference between a direct and indirect energy gap is between 100 and 200 meV. The linear region is clearer and more evident for the case that a direct transition is assumed. Recently, the properties of the hematite were calculated by several ab initio techniques, showing an indirect edge and a direct edge, dozens of meV above, from the calculation of its band structure [57]. Meanwhile, the absorbance obtained from the same calculations shows the behavior of a direct absorption edge like these samples [54]. If both bandgaps are present, the absorption due to direct bandgap dominates because it does not involve interaction with phonons, so it is a transition much more probable and faster. A shift of the *E*_g_ to lower energy values from the closest sample to the precursor (x = 10 cm) to the furthest (x = 22 cm) can be observed; the *E*_g_ energy decrease and the sample gets darker. It may be related to a decrease in the *E*_g_ value with the increase of the film thickness. This tendency was already reported for α-Fe_2_O_3_ [21,58] and other semiconductor oxides [59].

Finally, the theoretical absorption was compared with the experimental absorption. This comparison can be seen in Figure S3 in the Supplementary Materials. A better agreement is obtained in the direct gap method, so it can be concluded the nature of the gap resembles more a direct transition than an indirect transition, although several works assign Fe_2_O_3_ an indirect nature [13,44,46,47,54].

### 2.3. Photoelectrochemical Properties of the Hematite Thin Films

**Figure 7:**
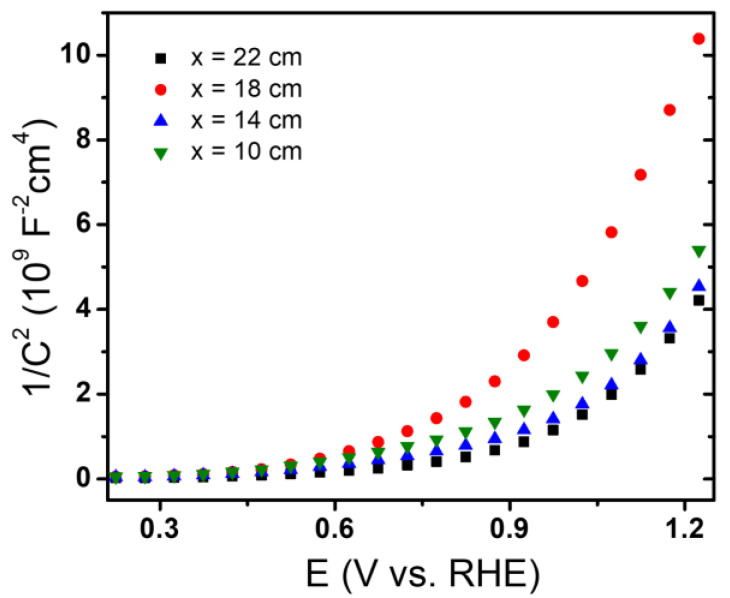
Mott–Schottky diagrams registered in NaOH 1 M, at 1 kHz. The diagrams correspond to samples prepared at the positions of 10, 14, 18, and 22 cm.

To study the semiconducting and photoelectrochemical properties of hematite films, they were characterized by Mott–Schottky and by linear scan voltammetry in the dark and under illumination. Due to the nanocrystalline morphology of the films, the simple Mott–Schottky equation may not be applicable. However, in a first approximation, this type of analysis can be applied to nanostructured systems. Examples of this approach to characterize nanostructured semiconductor thin films can be encountered in the literature [10]. The Mott–Schottky diagrams for α-Fe_2_O_3_ electrodes in 1 M NaOH solution measured at 1.0 kHz are shown in Figure 7. All samples exhibit similar behaviors where two regions can be defined, one with a dependence almost parallel to axis X, extended from 0.2 V to 0.6 V, approximately, and the other with a pronounced positive slope from 0.6 to higher potentials. From a linear fit of the data in the second region, the flat band potential and the apparent majority carrier density values, have been obtained from the extrapolation and the slope, respectively. For all samples, an n-type behavior was observed, and a value close to 0.8 V was found for the flat band potential (E_FB_). The obtained majority carrier density (ND) values are 4.55·10^21^, 5.08·10^21^, 2.27·10^21^, and 5.13 ·10^21^ cm^−3^ for samples fabricated at 10, 14, 18, and, 22 cm, respectively. It can be seen that all the values are in the order of magnitude of 10^21^, and only the sample prepared at 18 cm has a value close to half of the others. The values of flat band potentials and the density of majority carriers are similar to those reported in the literature [10,60]. In the case of flat band potential, several authors report that same value [1,7,10]; however, it is noteworthy that the density of majority load carriers is between 2 and 3 orders of magnitude higher than those reported by these authors which could be explained by the growth structure obtained by CVD [1].

**Figure 8:**
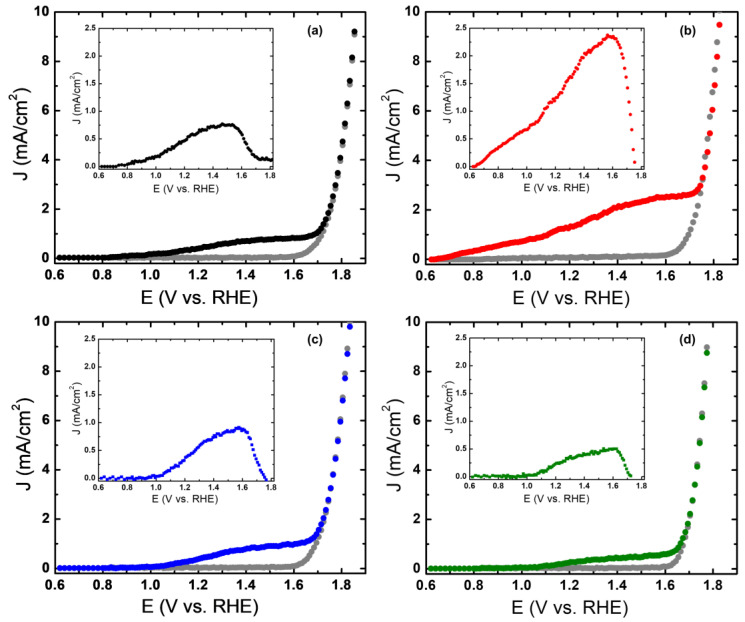
(**a**–**d**) are the current density-potential (J–E) characteristics of α-Fe_2_O_3_/FTO electrodes in 1 M NaOH in dark and under illumination for samples prepared at 10, 14, 18, and 22 cm, respectively. (Inset) The photocurrent-potential curve of α-Fe_2_O_3_/FTO electrode in 1 M NaOH.

In Figure 8, the potentiodynamic j/E profiles of the electrodes with and without white light illumination are shown. In this figure, it is possible to observe that in dark conditions, only capacitive currents are observed in all the potential range studied and from 1.6 V an anodic current is observed due to the water electrooxidation. The increase in the current density of the electrode observed under illumination confirms that the hematite film is photoelectrochemically active. Figure 8 shows the current density versus the potential for samples fabricated at 10, 14, 18, and 22 cm. The start of the photocurrent, that is, the potential at which the current density begins to increase is different for sample 18 being 0.76 V, whereas, for samples 10, 14, and 22, it is 1.20, 1.16, and 1.11 V respectively. Samples were determined by the percentage of photocurrent efficiency being 0.28, 0.50, 1.35, and 0.46% for the sample prepared at 10, 14, 18, and 22 cm, respectively. Differences in sample thicknesses may explain the difference in the percentages of photocurrent efficiency. When the light falls on the hematite film, and the electron–hole pair is generated, the electrons migrate to FTO and the holes at the surface [4,5,25]. When the film is very thin, the formation of a few excitons occurs, and when the thickness is too thick, the separation is limited by the long diffusion distance that the load carriers must travel. A calculation of the effective absorption (A_E_) was performed [27], which depends on the absorption of the material and the solar photons flux; therefore, a relationship can be observed between the calculation made and the efficiencies obtained, as shown in Table 2.

The insets of Figure 8a–d correspond to plots of the difference of the current density under illumination and darkness against the potential. It is clearly observed that sample 18 has a greater photocurrent at all potentials, particularly at 1.23 and 1.58 V, it presents 1.31 and 2.36 mA/cm^2^, respectively. The anodic character of the photocurrent indicates that the film exhibits photoactivity with n-type behavior [1,7,9,28]. The oxygen vacancies are the main defects in hematite films, and these are responsible for the n-type behavior, due to the electrons that are donated to the driving band [13].

## 3. Materials and Methods

The synthesis of the hematite was carried out in two steps, the first being the volatilization and decomposition of the ferrocene (dicyclopentadienyliron from Sigma Aldrich) used as a precursor in an Ar (99.999% purity) atmosphere at 500 °C, and subsequently, the formation of the hematite was obtained in an O_2_ (99.999% purity) atmosphere at 550 °C. Both processes were carried out for 30 min with a 200 sccm flow of the respective gas. 

Hematite films were characterized by a field-emission SEM (FEI-Quanta 250 FEG). Cross-section, top, and 45 degrees views of each sample were imaged. Raman spectroscopy was performed by using a Witec Alpha 300 equipped with a laser of 785 nm. XPS was performed by using a system from physical electronics (Versa Probe II), and the structural characterization of the hematite films was examined by X-ray diffraction (XRD) by using a PW1840 diffractometer (30 kV, 40 mA, Cu Kα radiation with λ = 1.5406 Å). The transmittance spectra of the samples were measured in equipment consisting of a 1000 W Xe lamp (ORIEL 6271) light source and an ORIEL 77,250 monochromator. The transmitted light was detected with a UDT 11-09-001-1 (100 mm^2^ wide-area UV-enhanced unbiased silicon detector). Mott–Schottky diagrams were determined in a three-electrode cell (counter electrode: Pt; reference: Ag/AgCl) using a 1 M NaOH electrolyte with potentiostat 604C from CH Instruments. The potentiodynamic j/E profiles were determined in a three-electrode cell (counter electrode: Pt; reference: Ag/AgCl) using a 1 M NaOH electrolyte with potentiostat EZstat-Pro from NuVant System Inc and a solar simulator 96,000 from Newport-Oriel Instruments equipped with an Air Mass 1.5 G filter.

## 4. Conclusions

In this work, hematite thin films were grown by the CVD technique by using ferrocene as an iron precursor. The influence of the distance between the precursor and FTO substrate position (in the CVD system), on the different properties of the hematite films, has been exhaustively studied. It was found that the thickness of the hematite films (which is a critical parameter in the photoelectrochemical performance), depends linearly on this precursor–substrate distance. XRD results showed that the obtained films exhibited a single phase of the rhombohedrally centered hexagonal structure of Fe_2_O_3_ (α-Fe_2_O_3_, hematite). The XRD study also revealed that the obtained hematite films exhibited a pronounced preferential orientation along the highly conductive (001) basal plane of hematite. All samples are polycrystalline, and the broadening of the diffraction peaks demonstrates the nanocrystalline character of nanostructured α-Fe_2_O_3_ layers. Raman and XPS characterization confirmed the XRD results, showing that the obtained phase was pure α-Fe_2_O_3_ hematite without the presence of other impurity phases. The UV–VIS spectra showed an absorption edge in the region between 500 and 600 nm. Both direct and indirect bandgap energies were found close to 2 eV, the indirect one being between 100 and 200 meV lower than the direct one. Both bandgap energies decreased when film thickness increased. All samples exhibited an *n*-type electronic conductivity type and majority carrier density (N_D_) values close to 10^21^ cm^−3^. It was observed that the thickness of the films has a great influence on the photocurrent efficiency, obtaining the best results for thicknesses of approximately 200 nm. In fact, photoelectrochemical studies give photocurrent efficiencies from 0.28% to 1.35%.

## Figures and Tables

**Table 1:** Optical bandgap values estimated assuming direct and indirect optical transitions.

Precursor-Substrate Distance Xsp (cm)	Direct Optical Bandgap (eV)	Indirect Optical Bandgap (eV)
22	2.16	2.00
18	2.18	1.99
14	2.21	2.08
10	2.31	2.12

**Table 2:** Values of Thickness, Effective Absorption (A_E_), and Photocurrent Efficiency.

Precursor-Substrate Distance x_sp_ (cm)	Thin-Film Thickness (nm)	A_E_ (%)	Eff. (%)
22	362	65	0.46
18	222	75	1.35
14	122	56	0.50
10	29	13	0.28

## Data Availability

L. Fernandez-Izquierdo is the depositary of all the data generated by the study.

## Supplementary Materials

The following supporting information can be downloaded at: https://www.mdpi.com/article/10.3390/molecules28041954/s1, Figure S1: Electrode photography (FTO with hematite). All samples have a reddish bricklike color, getting darker as distance from precursor (x) increases (samples prepared at the positions 10, 14, 18, and 22 cm); Figure S2: XPS spectra of Fe2p and O1s signals fitted by using the software Multipack of hematite films grown at 14, 18, and 22 cm; Figure S3: Comparison between theoretical absorption and the experimental one. From the estimated gap, a theoretical absorption coefficient was constructed using the method that was used for obtaining α^corr^. Starting from the linear fitting for determining the energy gap, an absorption coefficient was constructed for a direct and an indirect edge. Then the α^back^ was added to make the comparison; Table S1: Values of atomic percentages obtained by EDS of the elements present in the samples.
