# Supplementary material for: CVD Growth of Hematite Thin Films for Photoelectrochemical Water Splitting: Effect of Precursor-Substrate Distance on Their Final Properties
Source: Molecules. 2023 Feb 18;28(4):1954. doi: 10.3390/molecules28041954 (PMC9967862; doi:10.3390/molecules28041954)
Supplement: Supplementary file 1 [file molecules-28-01954-s001.zip › molecules-2200108-supplementary.pdf]

Supplementary Materials

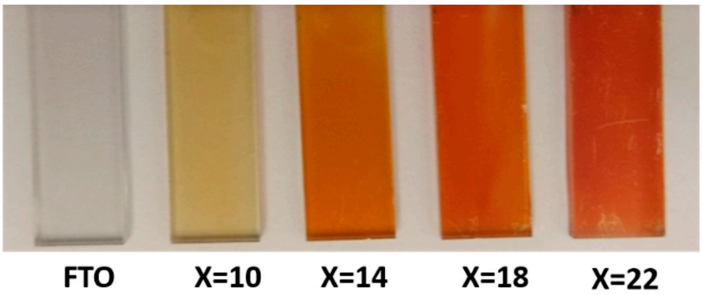

Figure S1: Electrode photography (FTO with hematite). All samples have a reddish bricklike color, getting darker as distance from precursor (x) increases (samples prepared at the positions 10, 14, 18, and 22 cm).

Table S1: Values of atomic percentages obtained by EDS of the elements present in the samples.

Element	Atomic %			
	FTO	x = 14 cm	x = 18 cm	x = 22 cm
O	43.88	54.13	59.90	62.72
Si	34.86	26.64	21.26	18.67
Sn	11.96	9.83	9.05	8.14
Ca	9.30	7.43	5.60	5.34
Fe	-	1.98	4.18	5.13

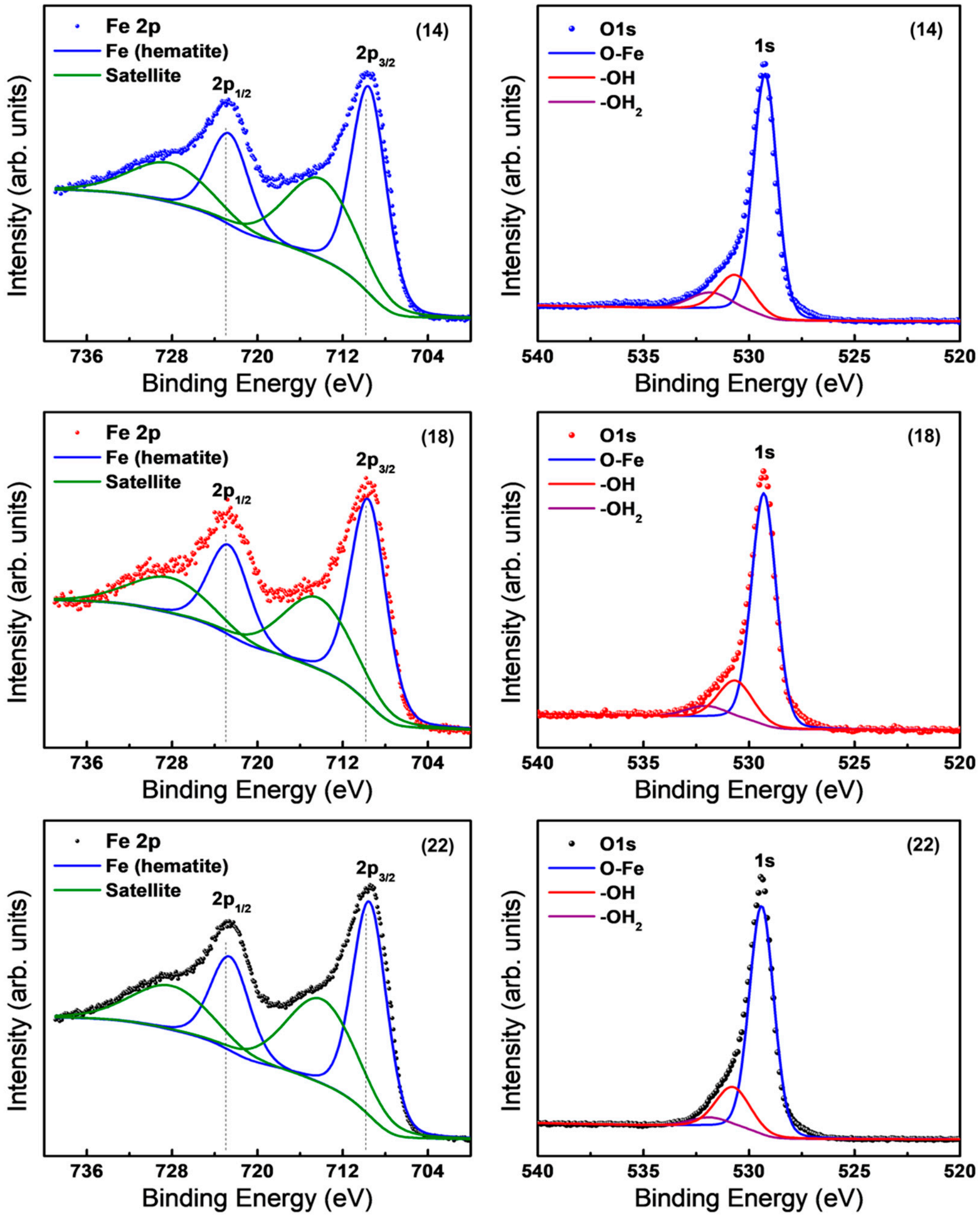

Figure S2: XPS spectra of Fe2p and O1s signals fitted by using the software Multipack of hematite films grown at 14, 18, and 22 cm.

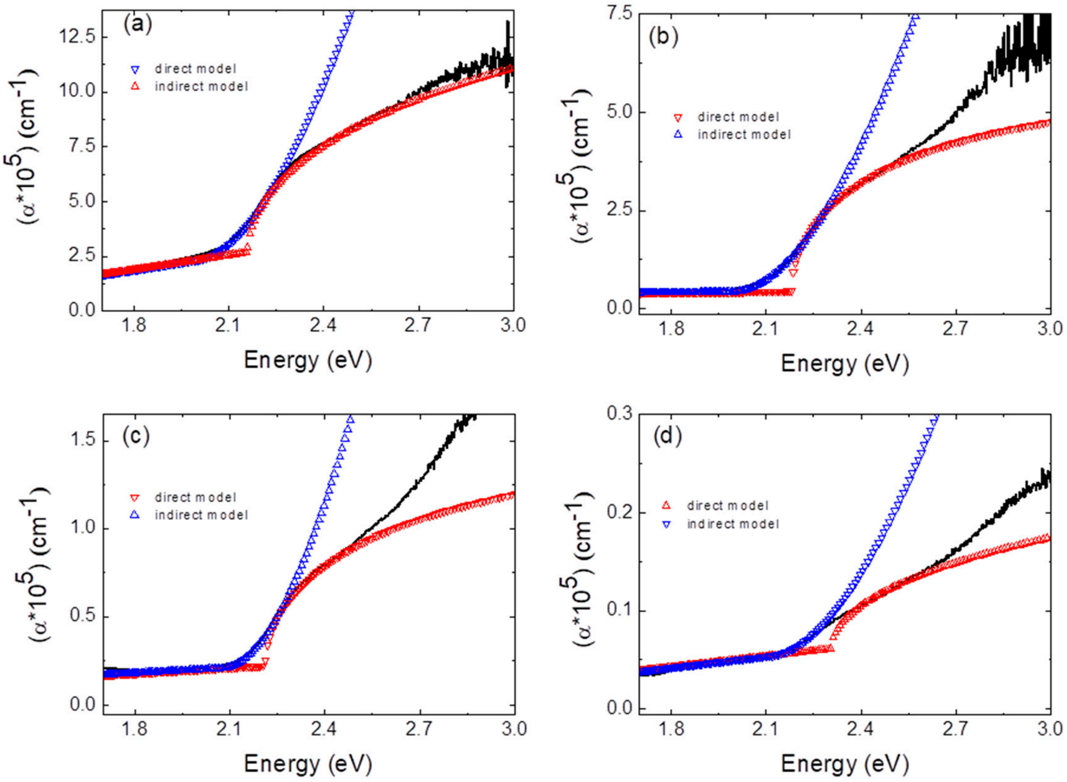

Figure S3: Comparison between theoretical absorption and the experimental one. From the estimated gap, a theoretical absorption coefficient was constructed using the method that was used for obtaining α_{corr}. Starting from the linear fitting for determining the energy gap, an absorption coefficient was constructed for a direct and an indirect edge. Then the α_{back} was added to make the comparison.
